# Editorial: Quality Control of Mammalian Oocyte Meiotic Maturation: Causes, Molecular Mechanisms and Solutions

**DOI:** 10.3389/fcell.2021.736331

**Published:** 2021-09-01

**Authors:** Yue Wang, Heide Schatten, Xiang-Shun Cui, Shao-Chen Sun

**Affiliations:** ^1^College of Animal Science and Technology, Nanjing Agricultural University, Nanjing, China; ^2^Department of Veterinary Pathobiology, University of Missouri-Columbia, Columbia, MO, United States; ^3^Department of Animal Sciences, Chungbuk National University, Cheongju, South Korea

**Keywords:** oocyte, meiosis, cell cycle, cytoskeleton, environmental exposure

## Introduction

The mammalian oocyte maturation quality is critical for successful fertilization and following early embryo development any errors will lead to birth defects, which might also cause infertility. The oocyte maturation includes two rounds of chromosome segregation and one round of DNA replication. Oocyte meiosis starts with germinal vesicle breakdown, and once a small polar body is extruded, the oocyte is arrested at metaphase II until fertilization. Previous studies have found that centrosome-mediated microtubule functions are important for the meiotic spindle organization in oocytes (Schatten and Sun, 2015) and that actin filaments drive spindle migration and cytokinesis during oocyte maturation (Duan and Sun, 2019), which is regulated by multiple factors such as Rab GTPases and Formins (Wang et al., 2019; Pan et al., 2020).

Recently, the effects of environmental exposure, diseases, and food safety on oocyte quality and maternal control have received more attention. It has been shown that environmental exposure for example to air, soil, water, and biota chemical pollutants, diseases like obesity and diabetes, or the intake of contaminated foods like mycotoxins all could affect oocyte maturation quality, reduce fertilization and early embryo development competence (Ou et al., 2019; Gallo et al., 2020).

This Research Topic, including seven reviews and 31 original research articles, offers critical and new insights into the causes and molecular mechanism of oocyte quality control, with particular insights into the cell cycle and cytoskeleton dynamics, mitochondria Ca2+ uptake, epigenetic modification, effects of environmental exposure, disease, and aging, and potential molecules for the protection of oocyte quality.

## Cell Cycle and Cytoskeleton Dynamics

Oocyte maturation is a complex and exquisite progression that includes two rounds of chromosome segregation and one round of DNA replication. The germinal vesicle breakdown and the first polar body extrusion are two critical processes during oocyte maturation, and the molecular mechanisms for regulating both GVBD and polar body extrusion are studied here. Wang and Pepling summarize the events and mechanisms controlling the progression through meiotic prophase one, which include recombination, synapsis, and control by signaling pathways.

Cyclins and cyclin-dependent kinases are important for the cell cycle. Among them, cyclin B1 is of particular interest because of its famous regulation function in oocyte maturation. Here, Cao et al. indicate that both CDK1 and ERK1/2 are required for the translational activation of Ccnb1 transcripts. However, CDK1 triggers phosphorylation, but not degradation of CPEB1 (cytoplasmic polyadenylation elements binding protein-1) in oocytes, and the degradation of CPEB1 was only triggered by ERK1/2. Li et al. found stable expression of another substrate of CDK1, cyclin B2, impeded segregation of sister chromatids after oocyte parthenogenetic activation, indicating that the cyclin B2/CDK1 complex conservatively regulates separase activity *via* inhibiting the phosphorylation of separase in mouse oocyte meiosis. In addition, the possible role of mechanotransduction and mechanosensor signaling pathways, namely the Hippo and the RhoGTPase, in the maturing oocyte is summarized by Pennarossa et al.

The post-translational modifications of tubulins are also key factors during cell cycle progression. Jeon and Oh found tumor suppressor that the RASSF1A (Ras-association domain family 1A) is required for tubulin acetylation by regulating SIRT2 and HDAC6 during meiotic maturation in mouse oocytes. Depletion of RASSF1A in oocyte caused abnormal spindle and misaligned chromosome, as well as disrupted kinetochore-microtubule attachment, and finally induced the increased incidence of aneuploid. Another member of the SIRT family, SIRT6, was also found to be critical in oocyte meiosis. Li et al. illustrate that inhibition of SIRT6 leads to meiosis arrest in porcine oocytes by inducing compromised spindle structure, misaligned chromosome, and impaired actin dynamics. Moreover, SIRT6 inhibition resulted in the defective cytoplasmic maturation by displaying the disturbed distribution dynamics of cortical granules and their content ovastacin. They also reveal that the meiotic defects caused by SIRT6 inhibition might result from the excessive reactive oxygen species (ROS) induced early apoptosis in oocytes.

## Mitochondria Ca2+ Uptake

Oxidative stress induces poor oocyte conditions. Calcium, especially that in the mitochondrial matrix, is strongly associated with oocyte activation disorders when mitochondria energy production is impaired by oxidative stress. Wang et al. used an encoded mitochondrial matrix Ca2+ probe (Mt-GCaMP6s) to observe mitochondrial Ca2+ ([Ca2+]_m_) dynamic changes during oocyte maturation and activation. They found that the active mitochondria surrounding the nucleus showed a higher [Ca2+]_m_ than those distributed in the cortex during oocyte maturation. During oocyte partheno-activation, the patterns of Ca2+ dynamic changes were synchronous in the cytoplasm and mitochondria. Their further results showed that higher [Ca2+]_m_ mitochondria around the chromosomes in oocytes might have a potential role in stimulating mitochondrial energy for calmodulin-responsive oocyte spindle formation while synchronizing Ca2+ functions in the cytoplasm and nuclear area are important for oocyte activation.

Since Ca2+ plays a critical role in both oocyte maturation and activation. Besides the roles of Ca2+ on mitochondrial energy supplementation, it is assumed that mitochondrial calcium homeostasis is involved in maintaining oocyte quality. Zhang et al. established a mitochondrial Ca2+ overload model in mouse oocytes by knocking down the gatekeepers of the mitochondrial Ca2+ uniporters Micu1 and Micu2 as well as the mitochondrial sodium-calcium exchanger NCLX in oocytes. They found the overload of mitochondria Ca2+ in oocytes impaired mitochondrial function, led to oxidative stress, and changed the protein kinase A (PKA) signaling associated gene expression as well as delayed meiotic resumption. Thus, the regulation of mitochondrial Ca2+ uptake in mouse oocytes has a significant role during oocyte maturation as well as PKA signaling. These results support a critical role for mitochondria Ca2+ during oocyte meiosis, and that proper mitochondrial Ca2+ reductions in obese oocytes might recover mitochondrial performance and improve oocyte quality.

## Epigenetic Modification

Epigenetic modification is also an important part of regulating oocyte maturation. Epigenetic modification during oocyte maturation is discussed in three review articles, including histone acetylation, phosphorylation, methylation, glycosylation, ubiquitination, and SUMOylation, which are highly related to oocyte quality control.

Wu et al. explored the function of maternal UHRF1 (ubiquitin-like, containing PHD and RING finger domains1) in zygotic genome regulation during early embryonic development in mice. Conditional knockout of UHRF1 in either primordial or growing oocytes causes infertility but differentially affects early embryonic development. UHRF1 deficiency in primordial oocytes led to early embryonic developmental arrest at the two-cell stage, accompanied by significant alterations in global DNA and H3K4me3 methylation patterns. In comparison, UHRF1 ablation in growing oocytes significantly reduced developmental competence from two-cell embryos to blastocysts. Cao et al. identified several novel and recurrent mutations of PATL2 (PAT1 homolog 2) in patients with a similar phenotype and chose a typical mutation to study the underlying mechanism. They found that this mutation decreased the ubiquitination level and degradation of PATL2, leading to an abnormal decrease in levels of Mos, and subsequently, it impaired the MAPK signal pathway and thus induced defects in oocyte maturation. Zhao et al. validated that a ubiquitin E3 ligases related protein, FBXO34, a member of Skp1–Cullin–F-box (SCF) complexes, plays a critical role in oocyte meiosis. Knocking down FBXO34 caused the failure of oocyte meiotic resumption due to the low activity of MPF, moreover, overexpression of FBXO34 promoted germinal vesicle breakdown (GVBD), but caused the MI arrest of oocytes. In addition, Wang et al. mentioned a Fibrillarin–GFP can serve as a marker for nucleolus activity, which also correlates with transcription activity and the quality of oocytes.

## Effects of Environmental Exposure on Oocyte Quality

The effects of environmental factors and toxins related to food safety on oocyte quality and maternal control have recently become a cause for concern. Here, a total of seven articles introduce the effects of different substance exposure on oocyte maturation, including Microcystin-leucine arginine, Particulate matter (diameter < 10 μM) (PM_10_), Bisphenol B, Ethylene glycol butyl ether, Paraquat, ferric ammonium citrate, and Copper (Cu), could disrupt the polar body extrusion, the meiotic spindle organization, and chromosome segregation. These exposed oocytes showed mitochondria dysfunction, relatively high levels of reactive oxygen species (ROS), DNA damage, apoptosis, and autophagy. These defects finally caused reduced oocyte maturation quality. In addition, Zhang et al. found that Bisphenol B exposure not only reduced oocyte quality by the ROS pathway, but also by changing the epigenetic modifications as indicated by increased K3K9me3 and H3K27me3 levels, and disrupting the pattern of estrogen receptor α (ERα) dynamics with a mass gathering on the spindle in BPB-exposed oocytes. Pan et al. also indicate that Bisphenol A exposure affects organelle function, *via* inducing mitochondria dysfunction, ER stress, abnormal structure of Golgi apparatus, and lysosome damage. All these defects may explain the reduced maturation quality of oocytes caused by substance exposure. Moreover, as parthenogenetic activation could be regarded as a method to reflect oocyte quality, Cui summarizes two types of oocyte spontaneous activation in a mini-review.

Toxic substances also reduce fertilization and early embryo development competence, which is further illustrated by Hu et al. and Xiao et al. Hu et al. showed that Podophyllotoxin exposure disrupted the organization of spindle and chromosome arrangement at the metaphase of the first cleavage in zygotes, induced excessive ROS, and caused embryonic DNA damage. Similarly, Xiao et al. found that Tributyltin oxide exposure also induced excessive ROS, caused mitochondrial dysfunction, disrupted cellular iron homeostasis and early apoptosis, which finally lead to the subsequent impartment of blastocyst formation.

## Effects of Disease and Aging on Oocyte Quality

Obesity and other diseases could also reduce oocyte quality. A mouse obesity model was established by Ge et al., in which telomere shortening was observed in both oocytes and early embryos from obese mice, as evidenced by the reduced expression of telomerase reverse transcriptase and activity of telomerase, indicating that telomere dysfunction might be a molecular pathway mediating the effects of maternal obesity on oocyte quality and embryo development. To explore the underlying mechanism for why EA (electroacupuncture) could prevent the progression of OHSS (ovarian hyperstimulation syndrome), and OHSS rat model was established and EA treatment was performed by Chen L. et al. They first identified that CD200 was a potential effector of EA in the ovary and elucidated the possible mechanism of EA in preventing and treating OHSS, which provides a scientific basis for CD200 as an effector and indicator in EA treatment.

For aging, Liu et al. investigated ASB7 (Ankyrin repeat and SOCS box 7) and found that it was critical for oocyte meiosis. Knocking down ASB7 induced abnormal spindle, misaligned chromosomes, loss of cortical actin cap, and impaired kinetochore-microtubule interaction. These further caused spindle assembly checkpoint activation, and lead to metaphase I arrest in the oocyte. They also found that aging oocytes normally had decreased expression levels of ASB7 and that increasing ASB7 expression can partially rescue the maternal age-induced meiotic defects in oocytes. Wang et al. found that the FKBP25 (FK506 binding proteins 25) protein level in old oocytes was also significantly reduced. They further found that oocytes with FKBP25 depletion display abnormal spindle assembly and chromosomes alignment, with defective kinetochore-microtubule attachment. Consistent with this finding, aneuploidy incidence is also elevated in oocytes depleted of FKBP25. The exogenous expression of FKBP25 could also partly rescue aging-associated meiotic defects. Moreover, they identify that serine 163 is a major phosphorylation site in modulating the action of FKBP25 during oocyte meiosis.

## Potential Solutions for Oocyte Quality Protection

Conducive items have also been suggested to maintain oocyte quality *via* different mechanisms. Zhang et al. indicated that vitamin C could protect oocytes by increasing the level of acetylate α-tubulin, maintaining actin polymerization, decreasing the level of cellular ROS, DNA damage, and early apoptosis, thus rescuing the meiosis arrest induced by Microcystin-leucine arginine exposure. Miao et al. reported that nicotinamide mononucleotide supplementation could rescue the meiotic defects caused by EGBE exposure *via* restoring NAD+ level and mitochondrial function and thus eliminating the excessive ROS. In addition, Mogroside V was demonstrated to promote oocyte maturation and embryonic development by Yan et al. Mogroside V can also protect oocytes from lipopolysaccharide-induced meiotic defects by in part oxidative stress and maintaining m6A levels. In addition, the role of lipids in controlling oocyte development was summarized by Khan et al. Moreover, Park et al. showed luteolin work as an antioxidant and could improve porcine meiotic progression and subsequent embryonic development by increasing the expression level of MOS, CDK1, and CCNB1, decreasing intracellular ROS levels and increasing blastocyst formation rate, as well as alleviating defects in cell organelles. Luo et al. showed that another antioxidant, imperatorin, was able to effectively delay oocyte aging and help to maintain oocyte quality by reducing oxidative stress and protecting mitochondrial function. Furthermore, milrinone is useful in improving developmental competence in less competent oocytes during IVM and for proper nuclear reprogramming in the production of porcine cloned embryos by coordinating cytoplasmic and nucleus maturation, illustrated by Roy et al. As widely known, Melatonin could benefit the maturation of the oocyte, both Wang et al. (2021) and Zhu et al. investigated the mechanisms to explain melatonin's ability to improve oocyte quality. In brief, melatonin could modulate lipid metabolism in maturing oocytes through its receptors in cumulus cells, and reduce the intracellular concentration of cAMP *via* melatonin membrane receptor MT1.

Overall, the findings in this Research Topic help to expand understanding and provide new insights about the molecular mechanisms of oocyte quality control, including aspects of cell cycle, cytoskeleton dynamics, epigenetic modification, organelle function, environmental exposure, disease, and aging. The new information provided in this topic is beneficial and lays theoretical foundations for improving female fertility.

## Author Contributions

YW wrote the manuscript. HS, X-SC, and S-CS revised the manuscript. All authors approved the manuscript for publication.

## Conflict of Interest

The authors declare that the research was conducted in the absence of any commercial or financial relationships that could be construed as a potential conflict of interest.

## Publisher's Note

All claims expressed in this article are solely those of the authors and do not necessarily represent those of their affiliated organizations, or those of the publisher, the editors and the reviewers. Any product that may be evaluated in this article, or claim that may be made by its manufacturer, is not guaranteed or endorsed by the publisher.
